# Developing independent investigators for clinical research relevant for Africa

**DOI:** 10.1186/1478-4505-9-44

**Published:** 2011-12-29

**Authors:** Yukari C Manabe, Elly Katabira, Richard L Brough, Alex G Coutinho, Nelson Sewankambo, Concepta Merry

**Affiliations:** 1 Infectious Diseases Institute, Makerere University College of Health Sciences, Kampala, Uganda; 2 Division of Infectious Diseases, Department of Medicine, Johns Hopkins University School of Medicine, Baltimore, Maryland, USA; 3 Makerere University College of Health Sciences, Kampala, Uganda; 4 Trinity College, Dublin, Ireland

## Abstract

Sustainable research capacity building requires training individuals at multiple levels within a supportive institutional infrastructure to develop a critical mass of independent researchers. At many African medical institutions, a PhD is important for academic promotion and is, therefore, an important focal area for capacity building programs. We examine the training at the Infectious Diseases Institute (IDI) as a model for in-country training based on systems capacity building and attention to the academic environment. PhD training in Africa should provide a strong research foundation for individuals to perform independent, original research and to mentor others. Training the next generation of researchers within excellent indigenous academic centers of excellence with strong institutional infrastructure will empower trainees to ask regionally relevant research questions that will benefit Africans.

## 

Research capacity building has been highlighted as an important strategy to improving health, alleviating poverty, and achieving the Millennium Development Goals in developing countries [1]. It has been defined as, "an approach to the development of sustainable skills, organizational structure, resources and commitment to health improvement... to multiply health gains many times over"[2]. Definitions of research capacity building often make reference to individual and institutional development as part of the process of research capacity building. Sustainable capacity building in clinical research, defined as research with human subjects or samples from human subjects, will require the development of a supportive environment conducive to individual development [3,4]. The increasing number of medical research grants with funded capacity building components has highlighted the need for increasing clarity regarding graduate research training in Africa. Defining the goals and the existing gaps in expertise to achieve these goals should be a priority. Institutions in Africa should seize the existing funding opportunities to create harmonized programs with clear, uniform expectations, accountability, and mentoring to ensure the success of individual trainees across programs. Finally, systems to increase resources and opportunities for students should be created at all levels to fill the pipeline with the quality, depth and number of trainees who can research medical questions relevant to sub-Saharan Africa (SSA) and sustainably mentor the next generation. Herein, we examine the training at the Infectious Diseases Institute (IDI) as a model for in-country training based on systems capacity building and attention to the academic environment.

### In country PhD training at the IDI, Makerere College of Health Sciences

Within academic institutions in sub-Saharan Africa, a PhD is often needed to be promoted within the academic ranks. Opportunities for medical graduates to get a PhD have been limited and require protected time and financial resources. In the past, the most successful model for training PhD students has been training abroad at an affiliated institution under the mentorship of a researcher with known research linkages to the country that sent the students (Fogarty model). Alternatively, with the sandwich PhD (SIDA SAREC model), the majority of training and research occurs in the low-income country, however, periods of training time are spent abroad for supervision and required methodology and theory courses [5]. Although in the past, such programs may have led to brain drain, most of these trainees return since the employment opportunities may currently be better in Uganda given the global economic crisis.

Training at local, public universities or freestanding Institutes provides important in-country opportunities and can eliminate the need to travel overseas. The IDI is a non-governmental organization owned by Makerere University and housed within the College of Health Sciences in Kampala, Uganda with a mission to improve health through research, training, and clinical care. At the IDI, a generous multi-year grant from Gilead has funded the Sewankambo Scholarship which currently supports 4 PhD candidates and one post-doctoral fellow. Other programs through the Dutch government and the European Union such as INTERACT (Infectious diseases Network for Treatment and Research in Africa), European Developing Country Clinical Trials Partnership (EDCTP), Belgian government (VLIR), and the Health Research Board in Ireland, have also provided tools, skills training, and, in close collaboration with Makerere University College of Health Sciences and the IDI, strengthened research infrastructure. Since 2006, the Gilead program has provided stipends for protected time for research, research funds as well as funding for administrative structures for accountability and mentoring. In addition, this capacity building grant offers funding for 2-4 Master's students per year who receive structured training in research methods as well as mentoring support to "fill the pipeline." This model has been successful in building capacity as evidenced by academic outputs from the research trainees (Figure 1). The number of local individuals able to mentor trainees has been limited due to brain drain in previous decades, clinical and teaching commitments, and a relatively small number of PhD holders compared to the number of students initiating training. At the IDI, mentoring support from a dedicated group of international faculty built from the founders of the institute [6,7] and the growing, diverse array of international partners with projects at the IDI has supplemented the outstanding, indigenous, affiliated faculty mentors.

**Figure 1:**
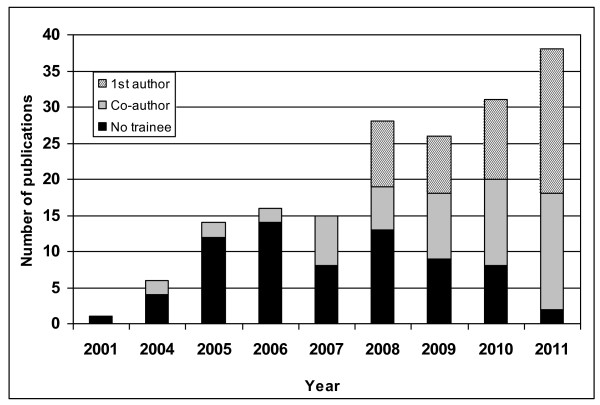
**Publications from the IDI since 2001**. The Institute opened in 2004 and funding for research capacity building at the IDI for PhD students began in 2006. An increasing number of trainee first-author (hatched bars), and co-authored (grey bars) compared to non-trainee authored peer-reviewed publications have been published at the IDI. 2011 bar represents publications published and in press as of September, 2011

### Systems Capacity Building

Through vigilant attention to individuals and systems, the IDI has been committed to sustainable capacity development in all three core areas, research, training and clinical care. The approach to building, and maintaining, research capacity is based on the recognition that various interdependent levels of capacity need to be strengthened for optimal results. These levels constitute a pyramid with, at the apex, the tools or "stock" which include equipment; next the skills through training (which is too often the extent of many research capacity building programs) which enable good use to be made of the tools; next adequate staff and facilities to meet continually assessed needs; and finally well-defined structures, roles, and systems which provide the necessary management foundation [3]. An additional level (the foundation layer) has recently been added to the capacity building pyramid called "local context," which highlights the need for durable capacity development to be based on recognition of cultural factors, alignment with local and national policies and strategies, trust between development partners, and local ownership. (Figure 2) Attention to each level gives individual researchers the best chance to excel and succeed. We applied this systems capacity building pyramid to research trainees, specifically PhD candidates. Within the context of the foundation layer of the model, local context, the IDI Research Department has instituted systems in the next layer to provide an environment conducive to research which include:

**Figure 2:**
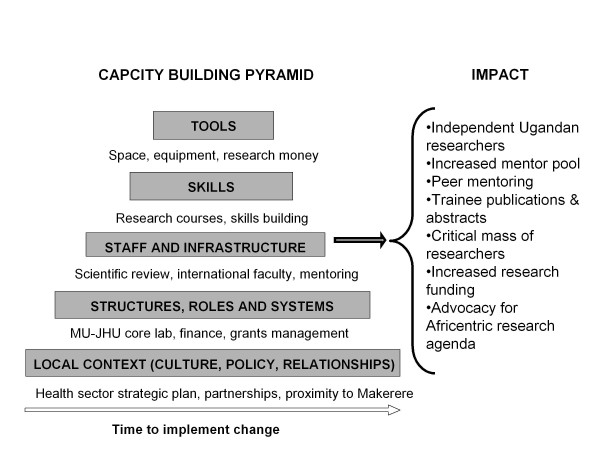
**The research capacity building pyramid inputs and impacts**.

1) rigorous scientific and operational review of all proposals at the IDI with a panel of investigators from within and outside the IDI

2) strong grants management and financial accountability

3) annual committee appraisals with clear performance metrics

4) clinical research training unit that offers regular trainings in good clinical practice and regulatory compliance, a new DataFax electronic data management system for local and remote use, and regulatory coordination and internal monitoring

5) strong, internationally accredited, core laboratory in support of clinical research with freezer repository storage.

### Challenges and Way Forward

Some critics have suggested that PhD programs in Africa cannot match similar programs in Europe or the US because of resource and institutional infrastructure constraints in Africa. For those students unable to spend time abroad, the PhD by publication has been the most attainable route to a PhD award that is required by many African universities for promotion to full professor. This type of PhD does not always guarantee that the individual has the capacity to generate research proposals or to perform research independently since a series of papers written in conjunction with others can be strung together for the thesis. In the end, what should an individual who has attained a PhD be able to do? We posit that Africans who have attained a PhD should have acquired knowledge in a specific area and the competencies to perform original research independently, have solid grounding in research methods both generically and in the specific area of the trainee's research and, ultimately, have gained the capacity to supervise others in lower cadres. Although published manuscripts remain the currency for academic productivity, promotion to full professor should not only be based on publications and a PhD, but also on high-quality, documented, mentoring of students. Linkages to a strong and functional research program like the IDI can provide the context to allow for continued growth beyond the PhD studies and opportunities to mentor [8].

PhD programs that are fully funded are still rare and lead to trainees choosing research areas opportunistically based on the funding rather than strategically according to their interests. This may be advantageous initially to develop a strong research foundation, but ultimately, research-training success should also be measured by trainees' pursuit of their own research interests driven by local needs. Formal training is time consuming and requires curriculum development and harmonization across capacity building programs given the limited number of mentors and teachers. Because the environment is less mature in terms of research methods courses and formal training of supervisors to mentor, the process cannot be wholly Darwinian for PhD students, but some success has to be guaranteed. This has to be balanced against the requirement for trainees to take responsibility for their own success; being chosen for a particular PhD opportunity cannot be synonymous with success. After PhDs are awarded, these students need to be further mentored within post-doctoral opportunities where they can solidify a fundable research focus and have grant writing mentorship. Finally, the issue of protected time for physicians pursuing a research career is difficult given the documented shortage of health care workers in Africa [9].

Consideration must also be given to the political context, the infrastructure at the university and/or research entity including grants management, data management, clinical laboratory, trained research coordinators, and most importantly to the number of qualified supervisors and their available time for mentoring as well as a broad range of expertise [10]. As Trostle pointed out, research requires funding, a research network, career path, personal and financial incentives, and political commitment [11]. Centers of Excellence such as the IDI can provide a research environment that can contribute to training a large group of researchers who may not have secured training opportunities such as those enumerated above. Clinical research platforms, short courses in research methods, lectures and other resources in kind has led to more than 40 Masters and PhD projects performed within the IDI for students who are funding their own education.

Centers for Global Health have become more commonplace at major universities in developed countries, and more faculty and students from the "North" are interested in pursuing medical research in resource-limited settings. As part of academic social responsibility [6], Northern faculty could identify promising African trainee candidates and provide individual mentoring. International faculty could also contribute to the development of junior faculty to improve their mentorship, research, and grant writing skills. Developing the infrastructure at African universities to build sustainable research programs and finding ways to raise the bar to the international level should become the goal; individuals must be trained to the level where they are able to mentor others. Institutions need to be strengthened in tandem to allow the efficient conduct of research. Centers for Global Health in Africa should be formed to harmonize international partnerships, and to insist on support and advocacy for institutional capacity building, including the development of the next generation of African researchers focused on the research needs and priorities of Africa. Francis Collins, the Director of the NIH, articulated five priorities for the NIH which included Global Health [12]. Recently, as evidence of the NIH commitment, collaborative funding from several US governmental agencies under the Medical Education Partnership Initiative was awarded to twelve African medical schools to strengthen medical institutions in Africa through partnerships with American universities. This funding has the potential to significantly enhance institutional infrastructure for African institutions and the opportunity for these institutions to plan strategically for the future rather than opportunistically apply for funding http://www.fic.nih.gov/programs/training_grants/mepi/index.htm.

In summary, training PhD students who can perform independent original research requires excellent well-funded programs within a strong environment with clear broad-based infrastructure. African academic medical centers should advocate for government support for Afro-centric clinical research and involve Ministries of Health early to jointly design research programs and to move research findings toward policy and practice. The success of these trainees should not only be measured by peer-reviewed published research productivity, but also by their ability to fund and execute research that will advance the health of Africans.

## Competing interests

The authors declare that they have no competing interests.

## Authors' contributions

The original concepts were developed by YCM, NS, EK, and CM. YCM drafted the manuscript in collaboration with RLB and CM. Extensive editorial support was given by AC, NS, and EK.
